# Inter-rater reliability of Hamilton depression rating scale using video-recorded interviews — Focus on rater-blinding

**DOI:** 10.4103/0019-5545.55085

**Published:** 2009

**Authors:** M. Krishna Prasad, K. Udupa, K. R. Kishore, J. Thirthalli, T. N. Sathyaprabha, B. N. Gangadhar

**Affiliations:** 1 Department of Psychiatry, National Institute of Mental Health and Neurosciences (NIMHANS), Bangalore – 560029, India; 2 Department of Neurophysiology, National Institute of Mental Health and Neurosciences (NIMHANS), Bangalore – 560029, India

**Keywords:** 21-item Hamilton depression rating scale (Ham-D), video recorded interviews, inter-rater reliability, rater blinding

## Abstract

**Background:**

Hamilton depression rating scale (Ham-D) is the most widely used clinician rating scale for depression. There has been no Indian study that has examined the inter-rater reliability (IRR) of video-recorded interviews of the 21-item Ham-D.

**Aim:**

To study the IRR of scoring video-recorded interviews for 21-item Ham-D.

**Materials and Methods:**

Eighteen subjects with major depressive disorder involved in a larger study were interviewed using the semi-structured clinical interview of the 21-item Ham-D by a primary rater after informed consent. These interviews were video-recorded and portions edited to ensure rater blinding. Subsequently, the video-recorded interviews were rated by a “blind” rater. Both rated the different sub-domains of Ham-D according to Rhoades and Overall (1983). IRR was evaluated using intra-class correlation coefficient.

**Results:**

Excellent IRR was observed (0.9891) between the two raters. This was true for each of the primary factors and super-factors.

**Conclusion:**

Video recorded 21-item Ham-D has excellentIRR. Video-recorded interviews of Ham-D can be reliably used to blind raters in research.

## INTRODUCTION

The term blinding (or masking) in medical research applies primarily to clinical trials. It is also commonly and increasingly used with reference to analytic epidemiologic studies.[1] The fundamental idea in blinding is that the study patients, the people involved with their management, and those collecting the clinical data from studies should not be influenced by knowledge of the assigned treatment or, in an epidemiologic study, by knowledge of the main risk factors or outcomes. Four levels of blinding have been described–single, double, triple, and quadruple.

Rater's knowledge of treatment assignment modifies the outcome assessment in clinical/epidemiological trials giving rise to rater bias. Raters may be unconsciously influenced by inclinations for or against a particular treatment and consequently may give a more or less generous assessment depending upon these biases.[2] Blinding of raters by keeping raters unaware of treatment assignment limits bias influencing assessment due to knowledge of treatment assignment.

There are many practical issues that pose difficulty in rater blinding. Several authors have challenged the effectiveness of rater-blinding itself.[1] For example, difficulty in blinding is conceivable in trials comparing typical antipsychotics and atypical antipsychotics, as the former cause extrapyramidal symptoms, which is often obvious. Similarly, tricyclic antidepressants induce dry mouth, and when compared to placebo, the question of which patients are on which treatment arm may not be difficult to guess. These issues have drawn some extreme (and perhaps debatable) argument that “all past studies of antidepressant effectiveness are open to question”.[3] Rater blinding is, however, such an important procedure in medical research that despite limitations one would have to use it. Several alternatives have been proposed. One such alternative is for a blind rater to rate videotaped interviews. This has been used in relation to many rating scales in psychiatry.[4]

Hamilton Rating Scale for Depression (Ham-D) was designed to measure the severity of depressive symptoms in patients with primary depressive illness.[5] It is by far the most frequently used clinician rating scale for depression.[6] It has excellent psychometric properties.[6] Many subsequent authors have attempted to derive meaningful factors from the original Ham-D.[7–10] Rhoades & Overall (1983)[11] arrived at a factor structure using the substantial collection of 21-item Ham-D from the New Clinical Drug Evaluation Unit data bank.

Hamilton found excellent correlation (0.9) between raters in his original paper. Others have found that Pearson's *r* ranged from 0.82 to 0.98, and the intraclass *r* ranged from 0.46 to 0.99.[12] This reliability has been found to hold good for raters using video-recorded interviews.[5] Psychiatric interviewing techniques and ratings are culturally sensitive. Hence, it is imperative that such exercises need to be replicated across cultures. No Indian study has examined the inter-rater reliability (IRR) of video-recorded Ham-D interviews.

### Aim

To study the IRR of scoring video-recorded interviews for the 21-item Ham-D.

## MATERIALS AND METHODS

The subjects for this study were part of a study examining the differential effects of three different antidepressant therapies on the heart rate variability in patients with major depressive disorder (MDD). The study included drug-naïve MDD patients referred to the study team by two clinical units of the psychiatry outpatient clinic of National Institute of Mental Health And Neuro Sciences (NIMHANS), Bangalore, India. Experienced psychiatrists confirmed the diagnosis according to DSM- IV TR[13] after clinical interview, physical examination, and pertinent laboratory investigations. The patients were 16- 50 years old (the study excluded patients older than 50 years), were of either sex, and had no other psychiatric or physical co morbidities.

Ninety-four patients were interviewed both at baseline and 1 month after starting antidepressant therapies, viz., rTMS, SSRIs, and TCAs. Eighteen interviews were video recorded. The mean (SD) age of the subjects was 33.83 years (8.25). Twelve (66%) of the recording were of male patients. Thirteen (72%) interviews were done in subjects before starting treatment and five interviews were after 4 weeks of antidepressant treatment. All interviews were in Kannada; the interviewer, blind rater, and the patients were all fluent in Kannada.

### Informed consent

The patients had already consented for the primary study. Additional written informed consent was obtained for recording their interviews using a video camera. Institute ethics committee approved this additional procedure.

### Hamilton depression rating scale (Ham-D)

A trained researcher (primary rater) administered the 21- item scale measuring depression before treatment and after completing antidepressant treatment or 1 month of drug therapy. The patients were interviewed using semi-structured interview for Ham-D[14] and the interview was video-recorded using a high-resolution digital camera with microphone. If, in the course of the interview, the subject revealed any information about his treatment status, those portions of the interviews were deleted before sending the videos to the “blind” rater. These video-recorded interviews were rated by a resident in psychiatry with 2 years of experience (“blind” rater).

The scores of the 21 items of Ham-D were organized into the different sub-domains of Ham-D according to Rhoades and Overall (1983)[11] for both raters. This included seven primary factors (somatization, diurnal variation, sleep disturbance, weight loss, depression, reality disturbance, and agitation/anxiety) and two super factors, which combined the seven primary factors. The super factors were vegetative depression (somatization, diurnal variation, sleep disturbance, and weight loss) and cognitive depression (depression, reality disturbance, and agitation/anxiety).

### Statistical procedures

Statistical tests were carried out in SPSS-10. IRR was evaluated using intra-class correlation (ICC) coefficient. ICC is a measure of reliability and is typically a ratio of the variance of interest to the sum of the variance of interest plus error.[15] It is used to measure the inter-rater and test-retest reliability of continuous constructs. As a rule of thumb, ICC above 0.8 is considered excellent, those in the range of 0.7-0.8 are considered good and those in the 0.5-0.7 range are considered fair.[16]

## RESULTS

The mean Ham-D scores (SD) were 14.00 (8.46) and 14.10 (7.75) for the primary and “blind” rater, respectively. The scores ranged from a minimum of 1 to a maximum of 26. Excellent IRR was observed (ICC coefficient: 0.9891) between the two raters. This was true for each of the primary factors and super factors. The ICC coefficients for total as well as sub-factors of Ham-D scale are given in Table 1.

**Table 1:** Intra-class correlation coefficient between the raters for the Ham-D factors

Ham-D super factors	Primary factors	Intra-class correlation coefficient
Vegetative depression	Somatization	0.9241*
	Diurnal variation	0.8329*
	Sleep disturbances	0.9457*
	Weight loss	0.8735*
	Total	0.9611*
Cognitive depression	Reality disturbances	0.8932*
	Mood depression	0.8565*
	Agitation/anxiety	0.7252*
	Total	0.8427*
Total Ham-D score		0.9891*

* *P* < 0.001

## DISCUSSION

### Inter-rater reliability

This study found that excellent IRR could be achieved using video recording of the Ham-D interviews by a “blind” rater. This was true not only with the total scores but also with the primary and super factors of the 21-item Ham-D. The interviewer used a semi-structured interview for Ham-D. Thus, all patients were asked the same set of questions, and where necessary, additional clarifying questions were asked. The total Ham-D score ranged from 1 to 26. This suggests that IRR was established across an appreciably wide range of severity of depression. The sample consisted of untreated as well as treated patients. Blinding to the treatment status was ensured by editing the videos to delete any information about the treatment status of the subjects.

Ham-D is commonly used in psychiatric research. Psychiatric interviews and assessments can be influenced by regional and cultural factors and need to be standardized to the respective regions. This is the first report of establishing IRR in India using video-recorded interviews for the 21-item Ham-D. This method has advantage in that the second rater could be effectively blinded for the treatment status of the subjects.

Establishing IRR using video-recorded interviews, however, does not entirely replace the need to establish the same of Ham-D using in-person interviews. This is because, Ham-D is rated using semi-structured interview. This leaves scope for variability introduced by the raters’ interviewing technique. Ratings given by two raters who interview the subject separately may thus show greater variability than that given using video-recorded interviews. Certain limitations of the study merit are mentioned here: the interviewers were aware that their interviews would be used for establishing IRR. This might have influenced the way they interviewed the subjects. Randomly selecting interviews from a larger number of interviews could partially avert this problem. The interviewer's style of interviewing may have biased the responses and the ratings as a semi-structured interview was used. Using a structured interview could avoid this, but the drawback of a structured interview is that while it achieves good reliability, it would compromise on validity. The video procedure does not eliminate but reduces the ‘openness’ of rating. The sample included 13 pre-treatment and 5 post-treatment interviews. A greater number of interviews including a higher proportion of interviews from post-treatment status would have enhanced the quality of the study. There is another potential psychometric issue that arises as to whether the ratings could be altered by the fact that the blind rater was not able to carry out the interview himself/herself. Comparing the ratings of one blind non interviewing rater on site during the interview and that of the blind rater subsequently would have addressed the issue.

### Merits of video-interview rating in psychiatric research

The importance of rater blinding in psychiatric research cannot be overemphasized. However, several practical factors prevent complete blinding and this affects the quality of the assessment. Assessment done using video-recorded interviews is an important alternative to actual assessment done by a blind rater. This confers added advantages[1] it can be used in situations where blinding is impossible, e.g., assessing the effectiveness of community-based programs. For instance, if a model of care is implemented in a community and its outcome is compared with that of a neighboring community in terms of burden on the family, the interviewer cannot be blind to the intervention status. Blinding can be introduced in such situations by video recording the interviews.[2] Treatment using complementary methods like Yoga and Ayurvedic procedures like shirodhara is increasingly becoming popular in psychiatric practice. Generally, trials using these procedures are single blind, as the subject cannot be blinded to the treatment mode. In such situations, the subject may inadvertently give away the procedure during an interview and break the rater blinding. Video-recorded interviews are advantageous in such situations since these can be carefully edited and information about treatment status can be deleted, thus keeping the rater blind to this information.[3] Some clinical trials in psychiatry are quite personnel intensive and hence are very expensive to conduct. For instance, trials using procedures like electroconvulsive therapy and transcranial magnetic stimulation will require separate research personnel to administer the treatment and to rate the subjects. Video-record based assessments may involve additional manpower in terms of the recording itself but can potentially obviate the need for employing separate fulltime raters solely for the purpose of blind assessment; several videos can be assessed in one sitting by part-time raters.[4] Over the sessions of assessment of the same patient to evaluate the outcome, a factor of expectancy effect[17] comes in. The rater inadvertently expects a change in the illness severity and may hence be giving different ratings over sessions of assessment. Video-recorded interviews with the date or order of interview blocked can reduce this effect. Several of the merits of the procedure such as the reduction of the bias stemming from expectancies and inadvertent sharing of group information flow logically from this procedure. The study demonstrates the IRR and validates this logic.

## CONCLUSION

This study found excellent IRR for the video- recorded interviews of the 21-item Ham-D for the first time in an Indian setting.
